# Health Care Use and Recurrence Rate in Hemolytic Disease of the Fetus and Newborn: Retrospective Cohort Study

**DOI:** 10.2196/88772

**Published:** 2026-06-05

**Authors:** Nehaa Khadka, Michael J Fassett, Carol Mao, Fagen Xie, Vicki Y Chiu, Jiaxiao M Shi, Theresa M Im, Sunhea Kim, Daniella Park, Darios Getahun

**Affiliations:** 1 Department of Research & Evaluation Kaiser Permanente Southern California Pasadena, CA United States; 2 Department of Obstetrics and Gynecology Kaiser Permanente West Los Angeles Medical Center Los Angeles, CA United States; 3 Department of Clinical Science Kaiser Permanente Bernard J Tyson School of Medicine Pasadena, CA United States; 4 Johnson & Johnson Horsham, PA United States; 5 Department of Health Systems Science Kaiser Permanente Bernard J Tyson School of Medicine Pasadena, CA United States

**Keywords:** hemolytic disease of the fetus and newborn, HDFN, alloimmunization, neonatal outcomes, recurrence, health care resource use, perinatal treatment, fetal anemia

## Abstract

**Background:**

Hemolytic disease of the fetus and newborn (HDFN) is a life-threatening condition resulting from maternal-fetal erythrocyte antigen incompatibility. Although anti–Rhesus D (RhD) prophylaxis has reduced RhD-associated cases, HDFN persists due to non-RhD antibodies and gaps in prevention. Population-based data on maternal and neonatal outcomes and recurrence patterns are limited.

**Objective:**

This study aimed to characterize maternal and neonatal outcomes, health care use patterns, and recurrence rates of HDFN across pregnancies.

**Methods:**

We conducted a retrospective cohort study of 464,711 pregnancies within the Kaiser Permanente Southern California system from January 1, 2008, to June 30, 2022. HDFN diagnoses were confirmed using validated natural language processing–assisted manual chart review and followed through 2023. Maternal characteristics, neonatal outcomes, and health care use were compared by HDFN status, and recurrence patterns were evaluated among individuals with ≥2 pregnancies. Chi-square tests and Wilcoxon rank-sum tests were used to compare characteristics between HDFN and non-HDFN pregnancies. Statistical significance was defined as *P*<.05.

**Results:**

Among all pregnancies, 139 of 464,711 (0.03%) were diagnosed with HDFN. Women with HDFN were more likely than those without HDFN to be older (aged ≥35 years; n=42, 30.2% vs n=97,146, 20.9%) and multiparous (n=121, 87.1% vs n=264,766, 57%). Infants affected by HDFN had higher rates of preterm birth (n=40, 28.4% vs n=42,240, 9.5%), low birth weight (<2500 g; n=22, 15.6% vs n=31,740, 7.1%), and neonatal jaundice (n=92, 65.2% vs n=162,465, 36.4%) than non-HDFN infants. Delivery hospitalizations (median 5.0, IQR 2.0-7.5 days vs median 2.0, IQR 1.0-2.0 days) and neonatal intensive care unit stays (median 4.0, IQR 0.0-7.0 days vs median 0.0, IQR 0.0-0.0 days) were longer, and maternal nondelivery hospitalizations were more frequent (n=27, 19.4% vs n=23,228, 5%) among pregnancies complicated by HDFN. Among women with a prior HDFN-affected pregnancy, 83.3% (n=25) experienced recurrence in a subsequent pregnancy. Of these recurrent cases, 32% (n=8) were severe, and 75% (n=6) involved fetal anemia requiring at least 1 intrauterine transfusion.

**Conclusions:**

HDFN was rare but was associated with substantial maternal and neonatal morbidity, including higher rates of preterm birth, increased neonatal intensive care unit admissions, and greater health care use. Recurrence was frequent and clinically significant, underscoring the importance of early surveillance and proactive management strategies.

## Introduction

Hemolytic disease of the fetus and newborn (HDFN) is a life-threatening condition caused by maternal-fetal erythrocyte antigen incompatibility, in which maternal immunoglobulin G alloantibodies cross the placenta and lead to fetal hemolytic anemia [1]. In severe cases, HDFN can result in fetal hydrops, fetal demise, and significant neonatal morbidity. The risk is particularly high for early-onset severe HDFN (≤24 weeks of gestation), which carries an elevated likelihood of stillbirth and adverse neonatal outcomes [2-4].

Widespread implementation of routine anti–Rhesus D (RhD) prophylaxis has substantially reduced RhD alloimmunization [5]. However, HDFN persists because of alloimmunization from non-RhD antigens or gaps in prophylaxis, highlighting ongoing challenges in prevention [5-7]. Current management for high-risk pregnancies includes middle cerebral artery (MCA) Doppler monitoring and intrauterine transfusions (IUTs) to prevent fetal hydrops and anemia-related pregnancy loss [8]. Although IUTs have improved perinatal survival [8], they remain invasive and carry procedure-related risks, including fetal demise, preterm birth, and other complications [2,3].

Alternative approaches, such as intravenous immunoglobulin (IVIG) and plasmapheresis, have been used to delay or reduce the need for IUTs, but outcomes remain suboptimal, and many affected pregnancies still require multiple procedures [8]. Beyond the immediate treatment challenges, recurrence of HDFN in subsequent pregnancies remains a major concern. Prior reports suggest that recurrence is common and may present with earlier onset or greater severity, yet robust epidemiological data remain limited [9].

A prior study suggested that maternal characteristics, along with medical and obstetric factors, may be associated with the diagnosis of HDFN [10]. Despite advancements in fetal monitoring and intervention, population-based evidence remains limited regarding whether HDFN impacts neonatal and maternal health care resource use. Furthermore, recurrence patterns in subsequent HDFN-affected pregnancies have not been well characterized within large integrated health systems. Therefore, this study aimed to (1) describe maternal and neonatal characteristics and health care use associated with HDFN and (2) evaluate recurrence patterns in subsequent pregnancies.

## Methods

### Ethical Considerations

All study activities were reviewed and approved by the Kaiser Permanente Southern California (KPSC) Institutional Review Board with exemption of informed consent (IRB#13503). The study involved a secondary analysis of existing electronic health record (EHR) data and posed no more than minimal risk to participants.

### Study Design

We conducted a retrospective cohort study using EHRs from KPSC, a large integrated health care system serving >4.7 million members across 15 hospitals and 235 medical offices. The KPSC population is racially, ethnically, and socioeconomically diverse and is representative of the Southern California population [11-13]. The KPSC database contains detailed sociodemographic, outpatient, inpatient, medical, and obstetric records, as well as pharmacy and laboratory records.

### Study Population

**Figure 1:**
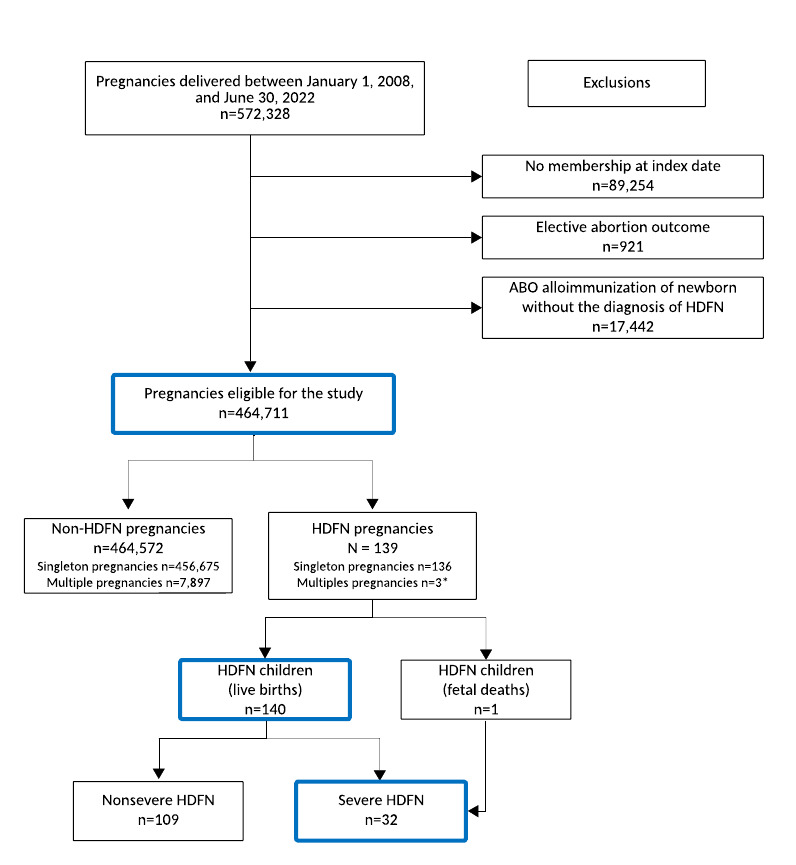
Study cohort composition. *Included 3 twin pregnancies, of which 5 infants had hemolytic disease of the fetus and newborn (HDFN), and 1 infant did not have HDFN.

Among 572,328 pregnancies delivered between January 1, 2008, and June 30, 2022, we excluded pregnancies that did not have KPSC membership at the start of pregnancy (n=89,254, 15.6%), those that ended in elective abortion (n=921, 0.2%), and cases with ABO alloimmunization without a confirmed HDFN diagnosis (n=17,442, 3%). The final analytic cohort included 464,711 (81.2%) pregnancies (Figure 1).

### HDFN Status and Classification

HDFN cases (from 2008 to 2022) were identified using a validated algorithm incorporating the *International Classification of Diseases*, *Ninth and Tenth Revisions*, *Clinical Modification* diagnostic and procedural codes, laboratory records, and clinical free-text extraction from KPSC EHRs and were followed through 2023. Trained abstractors conducted comprehensive chart reviews to confirm HDFN status, severity, and recurrence, with uncertain cases adjudicated by our maternal-fetal medicine specialist (MJF). Details of our HDFN identification process, including the natural language processing (NLP)–assisted chart review process, have been published previously [14].

Using structured EHR data (codes, medications, and laboratory results) and NLP-processed notes, candidate HDFN pregnancies were identified using likely indicators of maternal antibodies or titers; maternal or infant HDFN diagnosis, transfusion, or hydrops; infant IVIG; jaundice or phototherapy; and first Rho(D) immune globulin. We adjudicated selected indicator combinations by chart review and excluded cases of ABO alloimmunization alone. Of 464,711 eligible pregnancies, 136 (0.03%) were confirmed as HDFN. Indicator prevalence ranged from 0.02% (n=72; infant IVIG) to 34.53% (n=160,456; jaundice or phototherapy) overall and from 32.35% (n=44; infant IVIG) to 100% (n=136; maternal antibody positivity) among cases. Several combinations of 4 to 6 indicators had 100% positive predictive value; 80.88% (n=110) of cases had maternal antibody positivity, maternal or infant HDFN diagnosis, and infant jaundice or phototherapy.

### HDFN in Subsequent Pregnancies

We identified subsequent pregnancies among women with ≥2 pregnancies in the KPSC health care system between January 1, 2008, and December 31, 2023. Then, we ordered all pregnancies chronologically using the pregnancy episode start date, with the earliest pregnancy during the study period designated as the index pregnancy. All later pregnancies for the same individual were classified as subsequent pregnancies. For each subsequent pregnancy, HDFN status was determined using the same NLP-assisted manual chart review algorithm applied to index pregnancies.

### Maternal and Neonatal Characteristics

Maternal characteristics included age, race or ethnicity (non-Hispanic White, non-Hispanic Black, Hispanic, non-Hispanic Asian or Pacific Islander, other or multiple, or unknown), median household income (US <$30,000, US $30,000-$49,999, US $50,000-$69,999, US $70,000-$89,999, US ≥$90,000, or missing) based on US Census Bureau data, and insurance type (Medicaid, commercial, private, or other). Smoking, alcohol, and substance use during pregnancy were assessed, along with reproductive history (parity, gravidity, and prepregnancy BMI [kg/m²]). Clinical conditions included gestational weight gain (in pounds) and maternal comorbidities, such as asthma, chronic hypertension, pregestational diabetes, renal disease, and autoimmune disorders. We also evaluated obstetric complications, including preterm premature rupture of membranes, small-for-gestational-age or intrauterine growth restriction, and fetal hydrops.

Neonatal characteristics included infant sex, birth weight (grams), gestational age at birth (weeks), and head circumference (centimeters). Perinatal outcomes assessed were preterm birth, fetal death, and Apgar scores <7 at 5 minutes. Neonatal complications evaluated included birth asphyxia, hypoxic-ischemic encephalopathy, jaundice, kernicterus, and cerebral palsy.

### Health Care Resource Use

Health care resource use was evaluated separately for pregnant women and neonates. For pregnant women, we examined hospital length of stay, intensive care unit admission, and the timing of prenatal care initiation. Health care encounters, including hospitalizations, emergency department visits, urgent care visits, and outpatient visits, were assessed from pregnancy through 6 weeks post partum. For neonates, we examined the length of delivery hospitalization, neonatal intensive care unit (NICU) admission and length of stay, and health care resource use from delivery through 1 year, including hospitalizations, emergency department visits, urgent care visits, and outpatient visits.

### Treatment Modalities

We evaluated both individual and combined treatment modalities among pregnant women, including IUTs, MCA Doppler ultrasounds, RhoGAM, antenatal steroids, magnesium sulfate, group B *Streptococcus* prophylaxis, and plasmapheresis. For neonates, treatment modalities included red blood cell transfusions, exchange transfusions, phototherapy for hyperbilirubinemia, and IVIG.

### Statistical Analysis

We analyzed maternal and neonatal characteristics, health care resource use, and treatment patterns according to HDFN diagnosis status. Recurrence of HDFN was examined by estimating the frequency and proportion of pregnancies with a subsequent HDFN-affected pregnancy, including classification by severity and onset. Among recurrent HDFN cases, we described the distribution of gestational age at first IUT and summarized associated clinical features, such as fetal anemia and the number of IUTs performed. We excluded pregestational diabetes, birth asphyxia, hypoxic-ischemic encephalopathy, and cerebral palsy from all analyses due to the small sample size.

For categorical variables, frequencies and percentages were estimated, and comparisons were made using chi-square tests or Fisher exact tests when cell sizes were small. For continuous variables, means and SDs were calculated and compared using Wilcoxon rank-sum tests to account for potentially nonnormally distributed characteristics. Statistical significance was defined as a 2-sided *P* value <0.05. All analyses were conducted using SAS (version 9.4; SAS Institute Inc).

## Results

### Pregnancy Cohort

Among 464,711 singleton and multiple pregnancies in KPSC members between January 1, 2008, and June 30, 2022, 139 (3 per 10,000) were identified as HDFN pregnancies. There were 3 twin pregnancies, for which all but 1 fetus was diagnosed with HDFN, resulting in 140 HDFN live births and 1 HDFN fetal death (Figure 1).

### Maternal Characteristics

Women with HDFN pregnancies were older (aged ≥35 years; n=42, 30.2% vs n=97,146, 20.9%; *P*<.007) and had varied racial or ethnic distributions, with a higher proportion of non-Hispanic White women (n=55, 39.6% vs n=125,971, 27.1%) and a lower proportion of Hispanic women (n=57, 41% vs n=213,468, 45.9%; *P*=.005; Table 1**)**. Medicaid coverage was more common among HDFN cases (n=22, 15.8% vs n=44,561, 9.6%; *P*=.03), as was renal disease (n=8, 5.8% vs n=8331, 1.8%; *P*<.001). HDFN cases were also more likely to be multiparous (n=121, 87.1% vs n=264,766, 57%) and multigravida (n=128, 92.1% vs n=326,828, 70.4%; *P*<.001). Other sociodemographic, behavioral, and clinical characteristics were similar between groups.

**Table 1:** Distribution of maternal demographic, medical, and obstetric characteristics among pregnant women by hemolytic disease of the fetus and newborn (HDFN) diagnosis status.

Characteristics			Total pregnancies (N=464,711)		Pregnancies with HDFN (N=139)		Pregnancies without HDFN (N=464,572)		*P* value	
**Maternal age at index date (years)^a^**										<.001
	Mean (SD)	29.8 (5.7)		31.7 (5.3)		29.8 (5.7)				
	Median	30.0 (12.0-65.0)		32.0 (17.0-43.0)		30.0 (12.0-65.0)				
**Maternal age categories at index date (years), n (%)**										.007^b^
	<20	21,430 (4.6)		3 (2.2)		21,427 (4.6)				
	20-29	193,530 (41.6)		43 (30.9)		193,487 (41.6)				
	30-34	152,563 (32.8)		51 (36.7)		152,512 (32.8)				
	≥35	97,188 (20.9)		42 (30.2)		97,146 (20.9)				
**Maternal race or ethnicity, n (%)**										.005^b^
	Hispanic	213,525 (45.9)		57 (41.0)		213,468 (45.9)				
	NH^c^ Asian or Pacific Islander	62,045 (13.4)		17 (12.2)		62,028 (13.4)				
	NH Black	36,344 (7.8)		10 (7.2)		36,334 (7.8)				
	NH White	126,026 (27.1)		55 (39.6)		125,971 (27.1)				
	Other or multiple races	5794 (1.2)		0 (0.0)		5794 (1.2)				
	Unknown	20,977 (4.5)		0 (0.0)		20,977 (4.5)				
**Household income (US $), n (%)**										.80^b^
	<30,000	17,307 (3.7)		4 (2.9)		17,303 (3.7)				
	30,000-49,999	113,957 (24.5)		29 (20.9)		113,928 (24.5)				
	50,000-69,999	135,554 (29.2)		43 (30.9)		135,511 (29.2)				
	70,000-89,999	96,346 (20.7)		28 (20.1)		96,318 (20.7)				
	≥90,000	100,388 (21.6)		35 (25.2)		100,353 (21.6)				
	Missing	1159 (0.2)		0 (0.0)		1159 (0.2)				
**Insurance type, n (%)**										.03^b^
	Medicaid	44,583 (9.6)		22 (15.8)		44,561 (9.6)				
	Commercial	386,724 (83.2)		103 (74.1)		386,621 (83.2)				
	Private	27,385 (5.9)		12 (8.6)		27,373 (5.9)				
	Other	6019 (1.3)		2 (1.4)		6017 (1.3)				
**Smoking during pregnancy, n (%)**										.78^b^
	No	453,040 (97.5)		135 (97.1)		452,905 (97.5)				
	Yes	11,671 (2.5)		4 (2.9)		11,667 (2.5)				
**Alcohol use during pregnancy, n (%)**										.59^b^
	No	403,999 (86.9)		123 (88.5)		403,876 (86.9)				
	Yes	60,712 (13.1)		16 (11.5)		60,696 (13.1)				
**Drug use during pregnancy, n (%)**										.88^b^
	No	446,864 (96.2)		134 (96.4)		446,730 (96.2)				
	Yes	17,847 (3.8)		5 (3.6)		17,842 (3.8)				
**Prepregnancy BMI (kg/m^2^), n (%)**										.06^b^
	<18.5	9302 (2.0)		2 (1.4)		9300 (2.0)				
	18.5-24.9	172,300 (37.1)		52 (37.4)		172,248 (37.1)				
	25.0-29.9	121,422 (26.1)		28 (20.1)		121,394 (26.1)				
	30.0-34.9	68,732 (14.8)		25 (18.0)		68,707 (14.8)				
	≥35.0	54,829 (11.8)		12 (8.6)		54,817 (11.8)				
	Missing	38,126 (8.2)		20 (14.4)		38,106 (8.2)				
**Gestational weight gain (lb)**										.07^a^
	Mean (SD)	27.5 (15.9)		26.1 (16.2)		27.5 (15.9)				
	Median	27.6		24.0		27.6				
**Asthma, n (%)**										.38^b^
	No	440,344 (94.8)		134 (96.4)		440,210 (94.8)				
	Yes	24,367 (5.2)		5 (3.6)		24,362 (5.2)				
**Chronic hypertension, n (%)**										.05^b^
	No	455,405 (98.0)		133 (95.7)		455,272 (98.0)				
	Yes	9306 (2.0)		6 (4.3)		9300 (2.0)				
**Renal disease, n (%)**										<.001^b^
	No	456,372 (98.2)		131 (94.2)		456,241 (98.2)				
	Yes	8339 (1.8)		8 (5.8)		8331 (1.8)				
**Autoimmune disease, n (%)**										.31^b^
	No	463,441 (99.7)		138 (99.3)		463,303 (99.7)				
	Yes	1270 (0.3)		1 (0.7)		1269 (0.3)				
**Parity, n (%)**										<.001^b^
	Multiparous	264,887 (57.0)		121 (87.1)		264,766 (57.0)				
	Nulliparous	140,722 (30.3)		12 (8.6)		140,710 (30.3)				
	Unknown	59,102 (12.7)		6 (4.3)		59,096 (12.7)				
**Gravidity, n (%)**										<.001^b^
	Multigravida	326,956 (70.4)		128 (92.1)		326,828 (70.4)				
	Nulligravida	136,049 (29.3)		11 (7.9)		136,038 (29.3)				
	Unknown	1706 (0.4)		0 (0.0)		1706 (0.4)				
**Preterm premature rupture of membranes, n (%)**										.47^b^
	No	458,085 (98.6)		136 (97.8)		457,949 (98.6)				
	Yes	6626 (1.4)		3 (2.2)		6623 (1.4)				

^a^Wilcoxon rank-sum *P* value.

^b^Chi-square *P* value.

^c^NH: non-Hispanic.

### Neonatal Characteristics and Outcomes

Infants affected by HDFN had lower birth weight and gestational age distributions, with higher proportions born weighing <2500 g (n=22, 15.6% vs n=31,740, 7.1%; *P*<.001) and at 35 to 36 weeks’ gestation (n=24, 17% vs n=25,296, 5.7%) or 33 to 34 weeks’ gestation (n=12, 8.5% vs n=8753, 2%; *P*<.001; Table 2). Preterm birth was more common among HDFN cases (n=40, 28.4% vs n=42,240, 9.5%; *P*<.001). Head circumference was slightly smaller (mean 33.5, SD 2.26 cm vs mean 34.0, SD 2.72 cm; *P*=.04). Neonatal jaundice occurred more frequently in HDFN cases (n=92, 65.2% vs n=162,465, 36.4%; *P*<.001). Sex distribution and fetal death rates were similar between groups.

**Table 2:** Distribution of child characteristics and perinatal outcomes by hemolytic disease of the fetus and newborn (HDFN) diagnosis status.

Characteristics		Total births^a^ (N=446,499)	Pregnancies with HDFN (N=141)	Pregnancies without HDFN (N=446,358)	*P* value	
**Infant sex, n (%)**						.22^b^
	Female	216,646 (48.5)	59 (41.8)	216,587 (48.5)		
	Male	228,476 (51.2)	82 (58.2)	228,394 (51.2)		
	Unknown	1377 (0.3)	0 (0.0)	1377 (0.3)		
**Birth weight (g), n (%)**						<.004^b^
	<1500	5982 (1.3)	4 (2.8)	5978 (1.3)		
	1500-2499	25,780 (5.8)	18 (12.8)	25,762 (5.8)		
	2500-3999	366,607 (82.1)	113 (80.1)	366,494 (82.1)		
	≥4000	38,773 (8.7)	4 (2.8)	38,769 (8.7)		
	Unknown	9357 (2.1)	2 (1.4)	9355 (2.1)		
**Gestational age at birth (weeks), n (%)**						<.001^b^
	<28	3763 (0.8)	2 (1.4)	3761 (0.8)		
	28-32	6270 (1.4)	3 (2.1)	6267 (1.4)		
	33-34	8765 (2.0)	12 (8.5)	8753 (2.0)		
	35-36	25,320 (5.7)	24 (17.0)	25,296 (5.7)		
	≥37	402,381 (90.1)	100 (70.9)	402,281 (90.1)		
**Head circumference (cm)**						.04^c^
	Mean (SD)	34.0 (2.72)	33.5 (2.26)	34.0 (2.72)		
	Median (IQR)	34.0 (15.0-97.8)	33.7 (21.0-37.5)	34.0 (15.0-97.8)		
**Preterm birth, n (%)**						<.001^b^
	No	402,035 (90.0)	100 (70.9)	401,935 (90.0)		
	Yes	42,280 (9.5)	40 (28.4)	42,240 (9.5)		
	Missing	2184 (0.5)	1 (0.7)	2183 (0.5)		
**Fetal death, n (%)**						.71^b^
	No	444,315 (99.5)	140 (99.3)	444,175 (99.5)		
	Yes	2184 (0.5)	1 (0.7)	2183 (0.5)		
**5-minute Apgar score <7, n (%)**						.35^b^
	No	435,606 (97.6)	138 (97.9)	435,468 (97.6)		
	Yes	6083 (1.4)	3 (2.1)	6080 (1.4)		
	Missing	4810 (1.1)	0 (0.0)	4810 (1.1)		
**Neonatal jaundice, n (%)^d^**						<.001^b^
	No	283,942 (63.6)	49 (34.8)	283,893 (63.6)		
	Yes	162,557 (36.4)	92 (65.2)	162,465 (36.4)		
**Kernicterus, n (%)**						<.001^b^
	No	446,494 (100.0)	141 (100.0)	446,353 (100.0)		
	Yes	5 (0.0)	0 (0.0)	5 (0.0)		

^a^Includes 3 twin pregnancies, of which 5 children had HDFN and 1 child did not have HDFN (Figure 1).

^b^Chi-square *P* value.

^c^Wilcoxon rank-sum *P* value.

^d^This includes all neonatal jaundice with kernicterus cases.

### Health Care Resource Use in Pregnancy

Women with HDFN pregnancies had longer hospital stays (median 3.0, IQR 2.0-4.0 days vs median 2.0, IQR 1.0-3.0 days; *P*<.001) and initiated prenatal care later, with more patients beginning care in the second trimester (n=21, 15.1% vs n=23,175, 5%) or third trimester (n=5, 3.6% vs n=2477, 0.8%; *P*<.001; Table 3). Nondelivery hospitalizations were more common among HDFN cases (n=27, 19.4% vs n=23,228, 5%; *P*<.001). Urgent care visits were less frequent (n=23, 16.6% vs n=116,021, 25%; *P*=.02), while emergency department visits were similar between groups.

**Table 3:** Health care use among pregnant women based on hemolytic disease of the fetus and newborn (HDFN) diagnosis status.

Health care use			Total pregnancies (n=464,711)		Pregnancies with HDFN (n=139)		Pregnancies without HDFN (n=464,572)		*P* value
**Length of hospital stay during each pregnancy (days)**									<.001^a^
	Mean (SD)	2.4 (2.9)		3.6 (3.2)		2.4 (2.9)			
	Median (IQR)	2.0 (1.0-3.0)		3.0 (2.0-4.0)		2.0 (1.0-3.0)			
**Timing of prenatal care initiation, n (%)**									<.001^b^
	First trimester	398,818 (85.8)		110 (79.1)		398,708 (85.8)			
	Second trimester	23,196 (5.0)		21 (15.1)		23,175 (5.0)			
	Third trimester	3482 (0.8)		5 (3.6)		2477 (0.8)			
	No prenatal care or unknown timing	39,215 (8.4)		3 (2.2)		39,212 (8.4)			
**Health care use during each pregnancy, n (%)**									
	Hospitalizations (excluding delivery admission)	23,255 (5.0)		27 (19.4)		23,228 (5.0)		<.001^b^	
	Emergency department visits	107,560 (23.2)		35 (25.2)		107,525 (23.1)		.57^b^	
	Urgent care visits	116,044 (25.0)		23 (16.6)		116,021 (25.0)		.02^b^	

^a^Wilcoxon rank-sum *P* value.

^b^Chi-square *P* value.

### Neonatal Health Care Resource Use

Children with HDFN had longer delivery hospitalizations (median 5.0, IQR 2.0-7.5 days vs median 2.0, IQR 1.0-2.0 days; *P*<.001) and longer NICU stays (median 4.0, IQR 0.0-7.0 days vs median 0.0, IQR 0.0-0.0 days; *P*<.001) than those without HDFN (Table 4). NICU admission within 4 weeks of birth was more frequent among HDFN cases (n=85, 60.7% vs n=45,739, 10.3%; *P*<.001). Emergency department, urgent care, and outpatient visit frequencies were similar between groups.

**Table 4:** Health care use among children based on hemolytic disease of the fetus and newborn (HDFN) diagnosis status.

Health care use		Total live births (n=444,315)	Pregnancies with HDFN (n=140)	Pregnancies without HDFN (n=444,175)	*P* value	
**Length of delivery hospitalizations (days)**						<.001^a^
	Mean (SD)	2.9 (7.9)	7.4 (11.6)	2.9 (7.9)		
	Median (IQR)	2.0 (1.0-2.0)	5.0 (2.0-7.5)	2.0 (1.0-2.0)		
**Length of NICU^b^ stay during the delivery hospitalization (days)**						<.001^a^
	Mean (SD)	1.5 (8.2)	6.7 (12.5)	1.5 (8.1)		
	Median (IQR)	0.0 (0.0-0.0)	4.0 (0.0-7.0)	0.0 (0.0-0.0)		
**Health care use from delivery to 4 week postpartum, n (%)**						
	NICU stay	45,824 (10.3)	85 (60.7)	45,739 (10.3)	<.001^c^	
	Emergency department visits	17,579 (4.0)	4 (2.9)	17,575 (4.0)	.50	
	Urgent care visits	78,083 (17.6)	30 (21.4)	78,053 (17.6)	.23	
	Outpatient visits	424,789 (95.6)	130 (92.9)	424,659 (95.6)	.11	

^a^Wilcoxon rank-sum *P* value.

^b^NICU: neonatal intensive care unit.

^c^Chi-square *P* value.

### Treatment Modalities

**Figure 2:**
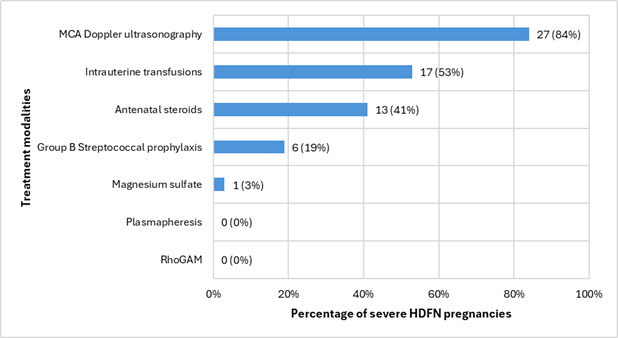
Distribution of treatment modalities among pregnancies with severe hemolytic disease of the fetus and newborn (HDFN; N=32). MCA: middle cerebral artery.

Figure 2 shows the frequency and percentage of treatment modalities for severe HDFN pregnancies (n=32, 100%). MCA Doppler ultrasound was the most frequently used modality among severe HDFN pregnancies (n=27, 84%), followed by IUT (n=17, 53%) and antenatal steroids (n=13, 41%). Group B *Streptococcus* prophylaxis was used in a smaller proportion of cases (n=6, 19%), while magnesium sulfate was rarely used (n=1, 3%). Plasmapheresis and RhoGAM were not used in this severe HDFN cohort.

**Figure 3:**
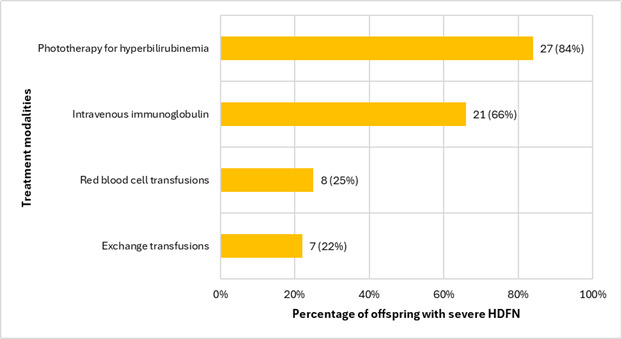
Distribution of treatment modalities among offspring with severe hemolytic disease of the fetus and newborn (HDFN).

Among offspring with severe HDFN, phototherapy for hyperbilirubinemia was the most common treatment (n=27, 84%), followed by IVIG (n=21, 66%). Red blood cell transfusions (n=8, 25%) and exchange transfusions (n=7, 22%) were used less frequently (Figure 3).

### Subsequent Pregnancies

Overall, 0.05% (53/101,623) subsequent pregnancies were affected by HDFN, with the majority occurring among those with a prior HDFN-affected pregnancy. The recurrence of HDFN in a subsequent pregnancy was substantially more common among pregnancies with a prior history of HDFN than among those without prior HDFN (12/16, 75% vs 41/101,607, 0.04%; *P*<.001).

Limiting the analysis to women with a prior HDFN-affected pregnancy who conceived again, recurrence was common: 83.3% (25/30) experienced HDFN in the next pregnancy, while 16.7% (5/30) did not (Table 5). Most recurrent cases were classified as nonsevere (n=17, 68%), although approximately one-third were severe (n=8, 32%). Among severe recurrences, onset occurred more often after 24 weeks of gestation (n=5, 62.5%). Evidence of fetal anemia was observed in 75% (n=6) of severe cases, necessitating at least 1 IUT.

**Table 5:** Hemolytic disease of the fetus and newborn (HDFN) status, severity, and management in subsequent pregnancies among women with a prior HDFN diagnosis.

Characteristics		Subsequent pregnancies, n (%)
**HDFN status of the subsequent pregnancy (n=30)**		
	Non-HDFN pregnancy	5 (16.7)
	HDFN pregnancy	25 (83.3)
**HDFN severity among recurrent cases (n=25)**		
	Nonsevere HDFN	17 (68)
	Severe HDFN	8 (32)
**Onset among severe cases (n=8)**		
	Early onset (≤24 weeks of gestation)	3 (37.5)
	Late onset (>24 weeks of gestation)	5 (62.5)
	Documented fetal anemia^a^	6 (75)
	≥1 intrauterine transfusion	6 (75)

^a^Fetal anemia was defined as a fetal hemoglobin level <0.84 multiples of the median. Fetal hemoglobin (g/100 ml) was calculated by dividing hematocrit (%) by 3.

## Discussion

### Principal Findings

In this large retrospective cohort study, we characterized maternal and neonatal outcomes, health care use, and recurrence patterns associated with HDFN. Although HDFN was rare, affected pregnancies demonstrated notable differences in clinical presentation and health care use, and recurrence in subsequent pregnancies was frequent.

### Maternal Characteristics, Neonatal Outcomes, and Health Care Use

HDFN pregnancies differed from unaffected pregnancies across several maternal and neonatal characteristics. Mothers with HDFN were older and more frequently multiparous, consistent with alloimmunization developing over multiple pregnancies. Neonates affected by HDFN had higher rates of preterm birth and low birth weight and were substantially more likely to require NICU care, with longer delivery and NICU hospital stays. These findings reflect the substantial burden of hemolysis-related anemia and hyperbilirubinemia, which often necessitate specialized postnatal care.

Health care use was elevated in pregnancies affected by HDFN. Women experienced more nondelivery hospitalizations and initiated prenatal care later, suggesting potential gaps in early detection and management. Neonates required significantly greater inpatient resources, amplifying the strain on NICU services. Together, these patterns underscore the complex care needs associated with HDFN and highlight the substantial burden on both obstetric and neonatal services.

### HDFN Recurrence in Subsequent Pregnancies

A key contribution of this study is the rates of HDFN recurrence. Among individuals with ≥2 pregnancies, 83.3% had HDFN in a subsequent pregnancy, and approximately one-third of recurrent cases were severe. Reported HDFN recurrence rates vary by cohort definition and outcome threshold. With recurrence defined as any HDFN in any subsequent pregnancy among women with a prior HDFN (EHR indicator or NLP phenotype) diagnosis, our rate is higher than the 56% treatment-requiring proportion reported among subsequent pregnancies of RhD-immunized women in Sweden [15], consistent with our higher-risk entry criterion and broader outcome. In severe cohorts, recurrence can be even higher: among women with a prior history of IUT and a subsequent antigen-positive fetus, 86% required repeat IUT [9]. Most recurrent cases demonstrated clinical features consistent with significant fetal anemia, including high frequencies of documented anemia and the need for IUTs. Recurrent cases that required IUTs often underwent treatment at earlier gestational ages than initially affected pregnancies, reflecting accelerated alloimmune fetal anemia in later pregnancies. These findings support current clinical observations that alloimmunization can lead to earlier and more clinically significant fetal disease in subsequent pregnancies. However, the presence of both severe and nonsevere recurrences suggests heterogeneity in disease expression.

### Clinical Implications

Our findings have important implications for antenatal counseling and management. Given both the high recurrence pattern and the potential for early or severe disease in subsequent pregnancies, enhanced surveillance strategies may be warranted. Early referral to maternal-fetal medicine, closer monitoring of antibody titers, and earlier MCA Doppler assessment may help identify high-risk pregnancies before significant fetal anemia develops. The observed variability in recurrence severity also supports individualized monitoring plans rather than uniformly escalating surveillance.

Furthermore, EHR-based algorithms could be developed to flag women at risk for HDFN recurrence at prenatal care intake, enabling early review of prior history, timely laboratory testing, and consideration of early intervention to optimize outcomes. Moreover, population-based studies are needed to clarify long-term neonatal outcomes and quantify the health care costs associated with HDFN across the perinatal period. Further research should also evaluate whether early maternal biomarkers or alloantibody trends can better predict severity or recurrence to guide the timing of surveillance and intervention. Studies of antenatal treatment strategies may help determine approaches that delay or reduce the need for IUTs. Finally, prospective multicenter investigations could validate these findings and explore long-term neurodevelopmental outcomes in affected neonates, addressing critical gaps in the current evidence.

### Strengths and Limitations

A major strength of this study is the large, demographically diverse, population-based cohort with comprehensive EHR data, enabling robust identification of HDFN cases and recurrence patterns. The manual chart review and adjudication process further strengthened data accuracy. However, our study is not without limitations. As this was a descriptive study, our findings should be interpreted accordingly. The retrospective design of this study may limit data capture in the EHR; therefore, misclassification of HDFN status, its severity, onset timing, and clinical features, such as fetal or neonatal anemia, is possible, particularly when documentation in health records was incomplete. Furthermore, future studies with larger samples and more events are needed to support multivariable regression modeling and adjustment for potential confounders. Although we used NLP-assisted manual chart review with adjudication to improve case ascertainment, cases from pregnancies managed in contracting hospitals (outside KPSC) may have been missed. This study was conducted within a single integrated health care system, which, while racially and socioeconomically diverse, may limit generalizability to nonintegrated health care settings.

### Conclusions

HDFN remains a clinically significant condition associated with substantial perinatal morbidity and elevated health care use. Recurrence in subsequent pregnancies was common, and many recurrent cases exhibited severe fetal anemia requiring early IUT. These findings underscore the importance of timely identification, targeted surveillance strategies, and continued research aimed at improving antenatal management and reducing the burden of disease.

## Abbreviations

EHR: electronic health record
HDFN: hemolytic disease of the fetus and newborn
IUT: intrauterine transfusion
IVIG: intravenous immunoglobulin
KPSC: Kaiser Permanente Southern California
MCA: middle cerebral artery
NICU: neonatal intensive care unit
NLP: natural language processing
RhD: Rhesus D
